# Advances in Genitourinary Tumor Genomics and Immunotherapy

**DOI:** 10.3390/genes16060667

**Published:** 2025-05-30

**Authors:** Jasmine Vohra, Gabriela Barbosa, Lívia Bitencourt Pascoal, Leonardo O. Reis

**Affiliations:** 1 Department of Biomedical Engineering, Vidyalankar Institute of Technology, Mumbai 400037, India; vohrajasmine07@gmail.com; 2 ImmuneOncology, Pontifical Catholic University of Campinas, PUC-Campinas, Campinas 13034-685, SP, Brazil; gabrielarbarbosa@hotmail.com (G.B.); livia.biazzo@puc-campinas.edu.br (L.B.P.); 3 INCT UroGen, National Institute of Science, Technology and Innovation in Genitourinary Cancer (INCT), Campinas 13083-970, SP, Brazil; 4 UroScience and Department of Surgery (Urology), School of Medical Sciences, University of Campinas, Unicamp, Campinas 13083-970, SP, Brazil

**Keywords:** genitourinary cancers, tumor genomics, cancer immunotherapy, immune checkpoint inhibitors, CAR-T-cell therapy, cancer vaccines, tumor microenvironment, liquid biopsy, artificial intelligence, multi-omics, personalized medicine, resistance mechanisms, microbiome, immune monitoring, precision oncology

## Abstract

Advancements in immune monitoring and modulation technologies are driving transformative changes in cancer immunotherapy. These innovations are crucial for assessing patient-specific immune responses, enabling more accurate predictions of therapeutic efficacy and enhancing treatment outcomes. This review provides a comprehensive overview of current technologies used in immune monitoring, such as flow cytometry, single-cell RNA sequencing, and multiplex cytokine profiling. It also explores cutting-edge immune modulation methods, such as biomaterials that activate immune cells and genetically engineered cell-based therapies. We examine the strengths and limitations of these techniques and identify areas where further progress is needed. In particular, we explore how personalized therapies, real-time monitoring systems, and artificial intelligence shape the future of immune-based treatments. Through a comparative analysis of existing platforms and emerging solutions, this paper underscores the importance of integrating diverse scientific approaches—from immunology and bioengineering to data science—in advancing safer, more effective cancer treatments. This interdisciplinary approach promises to enhance the precision and accessibility of immune-based therapies, offering new hope for improved cancer care.

## 1. Introduction

Genitourinary (GU) cancers—including those of the prostate, kidney, bladder, and testes—represent a major global health concern, with prostate cancer alone accounting for nearly 1.4 million new cases in 2020 [1]. These malignancies are biologically heterogeneous, with differences in tumor biology, genetic profiles, and therapeutic responsiveness, which necessitates nuanced clinical approaches [2,3]. Recent advances in next-generation sequencing (NGS) and single-cell omics have illuminated key molecular drivers. For instance, the discovery of Transmembrane Protease, Serine 2 gene (TMPRSS2) with the erythroblast R transformation gene (ERG) (TMPRSS2-ERG) fusions in over 50% of prostate cancers has enabled the stratification of patients and prediction of response to androgen deprivation therapies [4]. In clear cell renal cell carcinoma (ccRCC), mutations in von Hippel–Lindau (VHL), Polybromo 1 (PBRM1), and BRCA1 associated protein-1 (BAP1) have been linked with angiogenesis and immune infiltration patterns, influencing the efficacy of vascular endothelial growth factor (VEGF)-targeted and immune-based therapies [5].

Such findings support the broader vision of precision oncology, where treatments are tailored to the tumor’s mutational and immunological context. For example, bladder cancers with Fibroblast Growth Factor Receptor 3 (FGFR3) mutations have shown sensitivity to FGFR inhibitors like erdafitinib, illustrating how genetic data inform therapeutic choices [6]. Moreover, comprehensive molecular profiling from studies like the Cancer Genome Atlas (TCGA) has revealed immune subtypes across GU cancers, guiding immunotherapy decisions [7].

A transformative advance has been the integration of immune checkpoint inhibitors (ICIs). Trials such as IMvigor210 demonstrated durable responses to atezolizumab in programmed death-ligand 1 (PD-L1)–high-metastatic bladder cancer patients, though overall response rates remain modest [8]. Resistance mechanisms—such as T-cell exclusion or immunosuppressive microenvironments (TMEs)—continue to limit the breadth of response, necessitating biomarkers for better patient selection and therapeutic combinations.

To address these limitations, researchers are exploring integrated multi-omics analyses. For instance, work combined transcriptomic and immune data to define RCC immune subtypes, enabling tailored ICI regimens [9]. Additionally, emerging studies are investigating on-treatment biomarkers, such as T-cell clonality expansion, to monitor real-time immunotherapy effectiveness [10].

This review synthesizes key discoveries across genomics and immunotherapy in GU cancers, highlighting how mechanistic insights are reshaping precision treatment paradigms. 

## 2. Knowledge Evolution

Over recent decades, the landscape of GU cancer research has shifted from descriptive histopathology to deep molecular characterization. Landmark studies such as the TCGA Pan-GU analysis revealed distinct genomic architectures—like the high mutational burden (TMB) and apolipoprotein B mRNA editing enzyme catalytic polypeptide-like (APOBEC)-driven signatures in bladder cancer, versus copy number alterations and chromatin remodeling gene mutations in kidney cancers [11].

These insights have translated into novel clinical strategies. For example, the CheckMate 214 trial demonstrated superior overall survival with nivolumab and ipilimumab over sunitinib in metastatic RCC, especially in patients with intermediate/poor-risk disease—underscoring the role of immune gene signatures in predicting ICI benefit [12]. Similarly, the JAVELIN Bladder 100 trial established avelumab as maintenance therapy in advanced urothelial carcinoma, shifting the treatment paradigm based on immunotherapy responsiveness [13].

Biomarker development has been pivotal. In bladder cancer, high tumor mutational burden and (programmed death-1) (PD-L1) expression have been correlated with response to atezolizumab and nivolumab, although not all patients with high PD-L1 respond, highlighting the complexity of predictive modeling. Spatial heterogeneity that was revealed by multiplex imaging and spatial transcriptomics has further shown how T-cell localization within tumors affects ICI outcomes [14].

At the mechanistic level, immune evasion is increasingly understood through single-cell technologies. For instance, using single-cell RNA sequencing (scRNA-seq) in RCC to uncover T-cell exhaustion programs co-expressing PD-1, T-cell immunoglobulin and mucin-domain containing-3 (TIM-3), and lymphocyte activation gene 3 (LAG-3) led to trials targeting multiple checkpoints simultaneously. Such insights have also informed combination trials pairing ICIs with VEGF inhibitors or poly (ADP-ribose) polymerase (PARP) inhibitors, aiming to counteract tumor-intrinsic resistance pathways [15].

Artificial intelligence (AI) has emerged as a critical tool for integrating complex datasets. Deep learning models trained on histopathology slides and gene expression profiles have predicted ICI responsiveness with increasing accuracy, as seen in prostate and kidney cancer cohorts [16]. These approaches are not merely diagnostic but are being embedded in adaptive trial designs to inform real-time therapeutic decisions.

The TME has also gained prominence. In clear cell renal cell carcinoma (ccRCC), the presence of myeloid-derived suppressor cells and regulatory T-cells correlates with poor ICI response. Recent efforts to remodel the TME using oncolytic viruses, transforming growth factor β (TGF-β), inhibitors, or stimulator of interferon genes (STING) agonists aim to overcome this suppression and reinvigorate anti-tumor immunity [17].

Finally, the role of metabolism and epigenetics is being actively investigated. For example, fumarate accumulation in fumarate hydratase (FH)-deficient renal tumors drives immune evasion by altering the redox balance and impairing antigen presentation [18]. Epigenetic therapies, such as DNA methyltransferase (DNMT) or histone deacetylase (HDAC) inhibitors, are being trialed in combination with ICIs to reprogram “cold” tumors into immunologically “hot” ones [19,20].

In summary, the evolution of knowledge in GU cancers reflects a transition from histological categorization to an integrated view of genetics, immunity, and systems biology. This paradigm enables not only stratified therapies but also dynamic, patient-specific immuno-oncology interventions.

## 3. Genomic Landscape of Genitourinary Tumors

### 3.1. Prostate Cancer Genomics

Prostate cancer exhibits extensive molecular heterogeneity, driven by key genetic and epigenetic alterations that inform both clinical behavior and therapeutic strategies. One major category of molecular changes involves ETS gene fusions, particularly TMPRSS2-ERG, present in nearly 50% of prostate cancers and associated with distinct transcriptional programs [21]. In addition, mutations in speckle-type BTB/POZ protein (SPOP), a ubiquitin ligase substrate-binding protein, occur in about 10–15% of cases and are linked to genomic instability and sensitivity to DNA-damaging agents. TP53 mutations and phosphatase and tensin homolog (PTEN) loss are prevalent in advanced and castration-resistant diseases, correlating with treatment resistance and poor prognosis.

The androgen receptor (AR) pathway remains central to prostate tumor biology. Next-generation AR inhibitors like enzalutamide and abiraterone target ligand-dependent and -independent AR activation mechanisms, extending survival in metastatic castration-resistant prostate cancer [22]. However, resistance often develops through AR splice variants or intratumoral androgen synthesis. Emerging strategies include proteolysis-targeting chimeras (PROTACs) to degrade AR protein and dual inhibition approaches.

Defects in DNA damage repair (DDR) genes such as BRCA1, BRCA2, and ataxia telangiectasia mutated (ATM) represent actionable vulnerabilities. Olaparib and rucaparib, both PARP inhibitors, have shown efficacy in DDR-deficient tumors by inducing synthetic lethality [23,24]. For instance, the PROfound trial demonstrated significant radiographic progression-free survival in patients with BRCA1/2 or ATM alterations treated with olaparib [24].

Furthermore, chromatin remodeling and epigenetic modifications influence AR binding and transcriptional output. Aberrant DNA methylation and histone modifications (e.g., enhancer of zeste homolog 2 [EZH2] overexpression) contribute to lineage plasticity and neuroendocrine differentiation, posing therapeutic challenges [25]. Advanced sequencing technologies, including chromatin immunoprecipitation sequencing (ChIP-seq) and assays for transposase-accessible chromatin using sequencing (ATAC-seq), are being employed to map these changes.

Recent advances in liquid biopsies, particularly circulating tumor DNA (ctDNA) analysis, offer dynamic monitoring of tumor evolution and therapeutic resistance (Table 1). Studies have shown concordance between tissue and plasma genomic profiles, enabling non-invasive assessments of tumor burden and clonal shifts [26].

### 3.2. Renal Cell Carcinoma Genomics

Clear cell renal cell carcinoma (ccRCC), accounting for ~75% of RCC cases, is driven by biallelic inactivation of the VHL gene in over 90% of cases. The loss of VHL stabilizes hypoxia-inducible factors (HIF1α and HIF2α), which activate pro-angiogenic and metabolic pathways [27]. HIF2A-selective inhibitors like belzutifan are now under clinical development, directly targeting this axis [28].

Additional recurrent mutations in chromatin-modifying genes—PBRM1 (~40%), SETD2 (~15%), and BAP1 (~10%)—influence tumor aggressiveness and immune infiltration [29,30]. PBRM1 loss has been linked to increased sensitivity to immune checkpoint blockade, whereas BAP1 mutations correlate with poor prognosis.

Metabolic gene mutations, such as those in fumarate hydratase (FH) and succinate dehydrogenase (SDH), underlie aggressive RCC variants like hereditary leiomyomatosis RCC. These tumors exhibit pseudohypoxia and are metabolically reprogrammed, paving the way for novel metabolic therapies [31].

Immune heterogeneity plays a pivotal role in RCC outcomes. Transcriptomic profiling has identified immune-inflamed, immune-excluded, and immune-desert phenotypes. Epigenetic reprogramming with agents like HDAC inhibitors is under investigation to sensitize immune-cold tumors to immunotherapy.

### 3.3. Bladder Cancer Genomics

Bladder cancer encompasses a wide mutational spectrum of genetic changes, with mutations in FGFR3 (activating mutations in ~20%), TP53 (loss-of-function mutations in ~50%), and ERBB2 (amplifications in ~10%) being particularly common [32,33] (Table 1). These alterations are enriched in molecular subtypes defined by expression profiles: luminal tumors frequently harbor FGFR3 mutations and express uroplakin genes, while basal tumors exhibit squamous differentiation, higher immune infiltration, and TP53 mutations [34].

**Figure 1:**
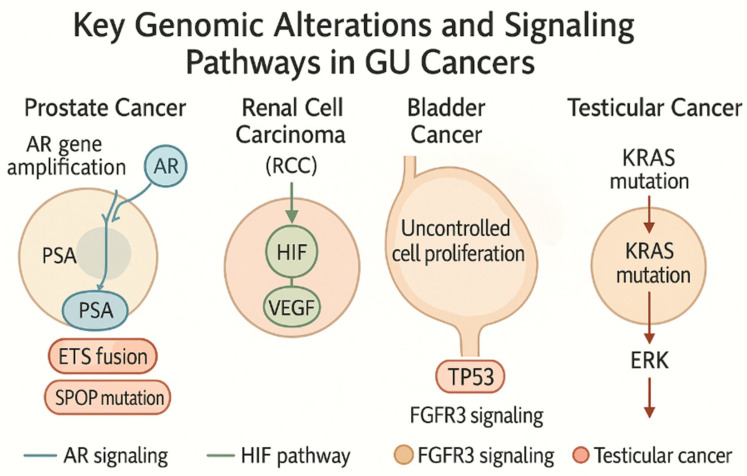
**Key genomic alterations and signaling pathways in genitourinary (GU) Cancers.** This illustration highlights major molecular drivers in GU cancers, including androgen receptor (AR) signaling in prostate cancer, hypoxia-inducible factor (HIF) pathway activation in renal cell carcinoma (RCC), FGFR3 (Fibroblast Growth Factor Receptor 3) signaling and TP53 alterations in bladder cancer, and KIT/KRAS mutations in testicular cancer. Created by the author with Official Adobe Photoshop—Photo & Design Software v. 25.12.

Targeted therapies have been developed accordingly. FGFR3-mutated tumors respond to FGFR inhibitors such as erdafitinib. The IMvigor210 trial highlighted durable responses to atezolizumab in basal-type tumors with high PD-L1 expression (Figure 1). This illustration of key genomic alterations and signaling pathways in genitourinary (GU) cancers highlights major molecular drivers in GU cancers, including androgen receptor (AR) signaling in prostate cancer, hypoxia-inducible factor (HIF) pathway activation in RCC, FGFR3 signaling and TP53 alterations in bladder cancer, and KIT proto-oncogene, receptor tyrosine kinase (KIT)/Kirsten rat sarcoma viral oncogene homolog (KRAS) mutations in testicular cancer.

Single-cell RNA sequencing has elucidated intra-tumoral heterogeneity, showing the co-existence of inflamed and immune-excluded regions within the same tumor mass. This heterogeneity is now being used to predict immunotherapy response and tailor combination regimens.

### 3.4. Testicular Cancer Genomics

Testicular germ cell tumors (TGCTs) arise from germ cell neoplasia in situ and diverge into seminomatous and non-seminomatous subtypes. KIT mutations and 12p chromosomal amplification (isochromosome 12p) are hallmarks of TGCTs, particularly in non-seminomas [35].

Epigenetically, seminomas maintain global hypomethylation, while non-seminomas show the promoter hypermethylation of tumor suppressors such as Ras association domain family member 1 isoform A (RASSF1A). These differences affect chemotherapy sensitivity and immune recognition [36]. Additionally, distinct microRNA profiles, including overexpression of the miR-371-373 cluster, serve as both diagnostic and prognostic biomarkers.

Resistance to cisplatin-based chemotherapy, which cures >90% of patients, is linked to the upregulation of DNA repair pathways (e.g., nucleotide excision repair (NER), mismatch repair (MMR)) and anti-apoptotic proteins [37]. Experimental treatments include immune checkpoint inhibitors and epigenetic drugs aiming to overcome resistance [38,39].

### 3.5. Emerging Trends in GU Cancer Genomics

Multi-omics approaches integrating genomics, transcriptomics, proteomics, and metabolomics are transforming GU cancer research. Liquid biopsies, which analyze ctDNA, CTCs, and extracellular vesicles, enable real-time disease tracking and early resistance detection [40]. Spatial transcriptomics and single-cell sequencing are exposing cellular niches, tumor–immune interactions, and therapy-induced remodeling. AI and machine learning algorithms are being deployed to decipher complex datasets, discover biomarkers, and predict drug responses [41]. Patient-derived tumor avatars, including organoids and xenografts, advance personalized therapy development by mimicking patient-specific drug responses ex vivo [42]. The microbiome is emerging as a modulator of cancer immunity. For instance, gut bacterial composition has been associated with differential ICI efficacy. Fecal microbiota transplantation (FMT) is being explored to restore responsiveness to immunotherapy. By integrating these platforms, precision oncology in GU cancers is progressing toward individualized, adaptive treatment paradigms [43].

## 4. Immunotherapeutic Approaches in Genitourinary Cancers

### 4.1. Immune Checkpoint Inhibitors

Immunotherapy, particularly through immune checkpoint inhibitors (ICIs), has ushered in a new era of genitourinary (GU) cancer treatment. ICIs work by blocking inhibitory pathways such as PD-1, PD-L1, and CTLA-4, which normally suppress T-cell activity, thereby enhancing the immune system’s ability to target cancer cells. Drugs such as pembrolizumab, nivolumab, and atezolizumab (Table 2) have demonstrated clinical efficacy, particularly in patients with advanced bladder cancer and RCC. The success of these therapies in other cancers has provided insights into their application in GU malignancies, though their effectiveness in prostate cancer remains limited [44].

For example, the KEYNOTE-045 trial revealed that pembrolizumab significantly improved survival in patients with advanced urothelial carcinoma, marking a major milestone for ICI therapy in bladder cancer. Similarly, nivolumab has been shown to enhance overall survival in RCC patients, though clinical response varies based on the presence of immune checkpoint markers. In prostate cancer, a higher tumor mutational burden (TMB) and microsatellite instability (MSI) have been associated with better responses to ICIs, underscoring the importance of patient selection and molecular profiling [45].

However, despite the breakthroughs, responses to ICIs are inconsistent. One of the key challenges is identifying reliable biomarkers that predict treatment outcomes. PD-L1 expression, TMB, and MSI have emerged as potential predictors of success, but their predictive value is still being refined in clinical trials. Ongoing research is investigating novel biomarkers, such as tumor-infiltrating lymphocytes (TILs), to stratify patients further and personalize treatment regimens. For example, a study explored the correlation between PD-L1 expression and clinical outcomes in RCC patients treated with nivolumab, showing a significant association between high PD-L1 expression and improved progression-free survival [46].

Furthermore, there is increasing interest in the role of the gut microbiome in modulating ICI efficacy. Recent studies have demonstrated that a diverse gut microbiome can enhance the effectiveness of ICIs, whereas dysbiosis may impair the immune response [47]. These findings open the door for microbiome-modulating therapies, potentially improving patient outcomes by restoring a beneficial microbial environment.

**Figure 2:**
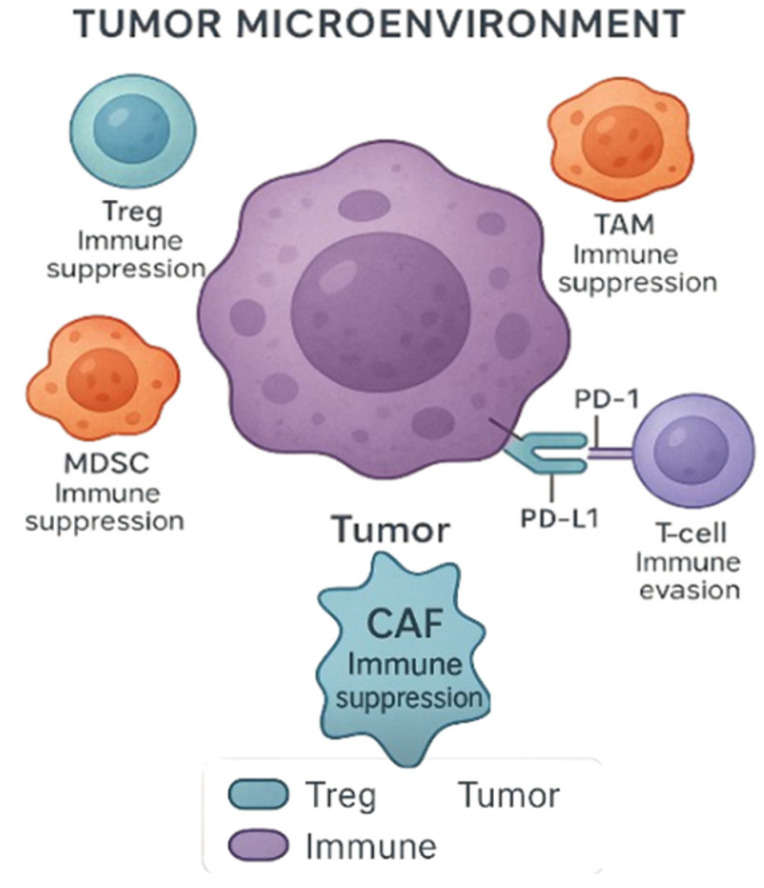
**Tumor microenvironment (TME) and immune evasion**. The TME includes various immune and stromal cells that suppress antitumor responses. Regulatory T-cells (Tregs), tumor-associated macrophages (TAMs), myeloid-derived suppressor cells (MDSCs), and cancer-associated fibroblasts (CAFs) contribute to immune suppression. CAFs are key stromal components known to modulate the immune landscape by promoting immune evasion and suppressing cytotoxic responses. Tumor cells can also evade immune surveillance through PD-L1 expression, which binds to PD-1 on T-cells, leading to T-cell exhaustion and impaired antitumor activity. Created with Official Adobe Photoshop—Photo & Design Software.

The TME is another critical factor affecting immunotherapy efficacy. GU tumors often create immunosuppressive microenvironments populated by regulatory T-cells (Tregs), myeloid-derived suppressor cells (MDSCs), and tumor-associated macrophages (TAMs), all of which suppress anti-tumor immunity (Figure 2) [48]. A recent study further elaborates on how these immune cell populations modulate the TME to evade immune surveillance, hindering the effectiveness of ICIs [49]. Strategies to overcome this resistance include combination therapies aimed at reprogramming the TME, such as using inhibitors of TGF-β signaling or myeloid cell depletion.

### 4.2. CAR-T-Cell Therapy and Adoptive Cell Transfer

Chimeric antigen receptor T-cell (CAR-T) therapy has emerged as one of the most innovative approaches in cancer immunotherapy, with remarkable success in hematologic malignancies. However, its application to solid tumors, including GU cancers, is still in its developmental phase due to several unique challenges. GU cancers often lack ideal surface antigens for CAR-T targeting, and their highly immunosuppressive TME limits T-cell activity [50] (Figure 2). Nevertheless, there have been significant strides in overcoming these barriers.

For instance, recent research introduced next-generation CAR-T-cells with modifications that allow for persistent T-cell activity and better infiltration of solid tumors. These engineered CAR-T-cells incorporate elements like cytokine receptors, which enhance their anti-tumor effects in hostile microenvironments. Moreover, the inclusion of checkpoint inhibitors, such as PD-1 inhibitors, directly into the CAR construct is an innovative approach currently under clinical testing [51]. Early-phase trials of these engineered CAR-T therapies have shown promising preclinical results in prostate cancer [52], with ongoing studies assessing their clinical efficacy.

In addition to CAR-T-cell therapies, other adoptive cell transfer methods, including tumor-infiltrating lymphocyte (TIL) therapy and T-cell receptor (TCR)-engineered cells, are being explored as potential treatments for GU cancers. A study highlighted the potential of TIL therapy in bladder cancer, showing that TILs can be expanded ex vivo and infused back into patients to stimulate anti-tumor immunity [53]. Furthermore, the combination of adoptive cell transfer with immunomodulatory agents like oncolytic viruses or immune checkpoint inhibitors has shown synergistic potential in preclinical models, suggesting a new frontier for treating GU malignancies [52,53] (Table 2).

### 4.3. Vaccine-Based Immunotherapy

Vaccine-based immunotherapies have gained considerable attention as a means of activating the immune system against cancer. These vaccines function by presenting tumor-specific or tumor-associated antigens to stimulate immune responses that target and destroy cancer cells. Sipuleucel-T, an autologous dendritic cell vaccine approved by the FDA for metastatic prostate cancer, represents one of the first successes in cancer immunotherapy [53]. However, the benefit of Sipuleucel-T is modest, and its use is limited to specific patient populations (Table 2).

Recent research efforts have focused on developing more tailored vaccine approaches, particularly those targeting neoantigens—mutations that are unique to an individual’s tumor. These neoantigen-based vaccines hold significant promise, as they exploit the tumor’s specific genetic makeup to generate a personalized immune response. A previous study demonstrated that a neoantigen vaccine induced robust immune responses and clinical benefit in patients with melanoma, and similar strategies are being investigated for prostate and bladder cancers [51].

Another promising avenue is the use of mRNA vaccines, which gained widespread attention during the COVID-19 pandemic. mRNA vaccines offer rapid customization and have the potential to target multiple tumor antigens simultaneously. Clinical trials are currently underway to adapt mRNA technology for cancer immunotherapy, including for GU cancers like prostate and bladder cancer [51]. Moreover, oncolytic viruses, which selectively infect and kill tumor cells while enhancing immune recognition, are being studied as novel vaccine delivery platforms. The combination of oncolytic viruses with ICIs could result in stronger and more durable immune responses, particularly in immune-resistant tumors [53].

This lists FDA-approved and investigational immunotherapies, their molecular targets, associated cancer types, and current clinical trial or approval status. These agents represent a shift in cancer treatment by enhancing the body’s immune response against tumors. Their use has expanded rapidly across GU cancers, especially in advanced or treatment-resistant cases.

## 5. Challenges and Limitations in Genomics-Driven Immunotherapy

### 5.1. Tumor Heterogeneity and Resistance Mechanisms

One of the most pressing obstacles in the application of genomics-driven immunotherapy for genitourinary cancers is the issue of tumor heterogeneity. Tumors are highly dynamic and evolve over time, leading to the emergence of multiple subclones with distinct genetic and epigenetic profiles. These subclonal variations can make it difficult to predict how a patient will respond to treatment. For example, in RCC, mutations in VHL (von Hippel-Lindau) and the phosphatidylinositol 3-kinase (PI3K) pathway have been shown to contribute to varying responses to ICIs. This genetic diversity within tumors complicates the identification of universal biomarkers and underscores the need for personalized therapeutic strategies. As tumors evolve, they often develop mechanisms of resistance to ICIs. Primary resistance occurs when tumor cells are intrinsically incapable of responding to the immune checkpoint blockade due to defects in antigen presentation, such as the loss of β2-microglobulin or mutations in JAK1/2. In contrast, acquired resistance arises after an initial response to therapy, when the tumor adapts by upregulating alternative immune checkpoints like TIM-3 or LAG-3 or by recruiting immunosuppressive cells like Tregs, MDSCs, and TAMs [53].

A noteworthy study investigated resistance mechanisms in metastatic melanoma, demonstrating that tumors can escape immune surveillance by downregulating MHC-I molecules, effectively preventing T-cell recognition. This phenomenon has also been observed in GU cancers, including bladder cancer, where similar immune evasion strategies are at play [54].

To tackle these challenges, advanced technologies like single-cell RNA sequencing and spatial transcriptomics are being employed. These techniques allow researchers to examine tumor heterogeneity in real time, providing deeper insights into how tumors evolve and how specific immune escape mechanisms, such as the loss of neoantigen expression or immune cell exclusion, contribute to resistance (Table 3). For instance, a study applied single-cell RNA sequencing to investigate intra-tumoral heterogeneity in bladder cancer, revealing distinct immune profiles that influence responses to ICIs [55].

**Figure 3:**
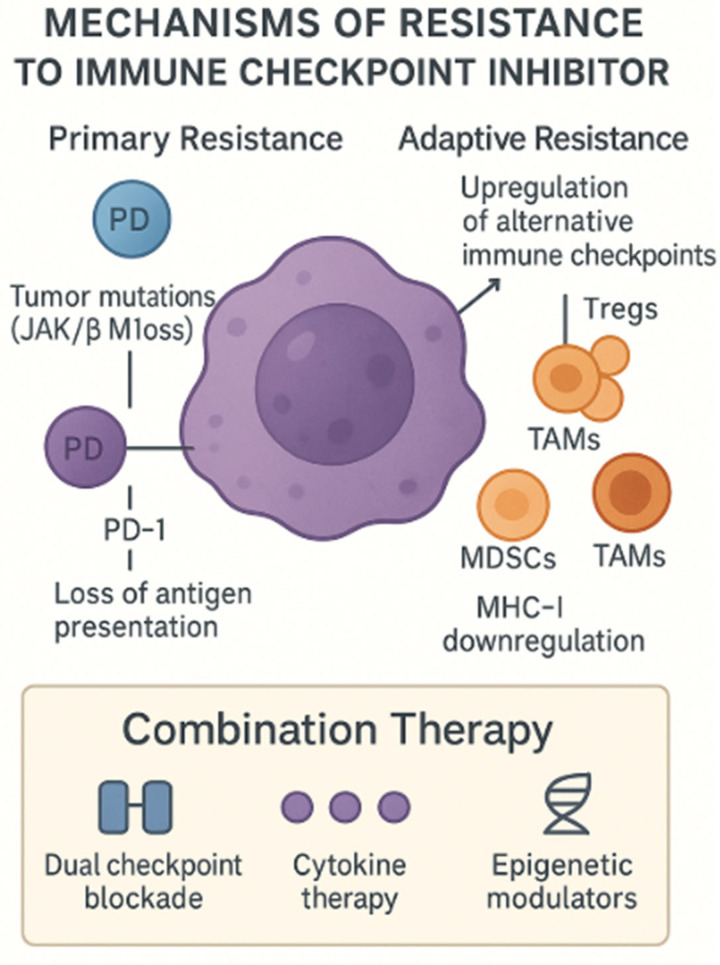
**Mechanisms of resistance to ICIs.** This schematic depicts both primary and adaptive resistance pathways by which tumors evade the checkpoint blockade. Primary resistance arises from tumor-intrinsic defects—such as JAK1/2 or β_2_-microglobulin loss—leading to impaired antigen presentation and T-cell exclusion. Adaptive resistance involves compensatory immune evasion strategies: the upregulation of alternative inhibitory receptors (e.g., TIM-3, LAG-3), the recruitment of immunosuppressive cells (Tregs, MDSCs (myeloid-derived suppressor cells), TAMs (tumor-associated macrophages)), and MHC-I (Major Histocompatibility Complex) downregulation. Combination approaches—dual checkpoint blockade, cytokine therapy, and epigenetic modulators—are under investigation to overcome these barriers. Created with Official Adobe Photoshop—Photo & Design Software.

Moreover, liquid biopsy technologies, such as ctDNA analysis, are emerging as non-invasive tools to track the evolution of resistance mechanisms. The various ways in which tumors evade immune responses—ranging from primary resistance due to the lack of antigen presentation to acquired resistance involving the upregulation of alternative checkpoints—are summarized in Figure 3.

### 5.2. Immune-Related Adverse Events

While ICIs have brought about significant clinical benefits, they are also associated with a unique spectrum of toxicities known as immune-related adverse events (irAEs). These toxicities can affect a wide range of organs, including the skin, gastrointestinal tract, endocrine glands, and lungs. Severe cases of irAEs, such as colitis or pneumonitis, can be life-threatening and may necessitate the cessation of immunotherapy. For example, a study reported a case of severe pneumonitis in a patient treated with pembrolizumab for metastatic melanoma, highlighting the potential risks of immune modulation [56]. Identifying patients who are at high risk for developing irAEs is a current research priority. Current studies are focusing on the role of specific biomarkers such as cytokine profiles, and HLA genotypes are being studied to predict irAEs. A study found that high levels of interleukin-17 (IL-17) were associated with an increased risk of developing immune-related dermatitis in patients receiving ICIs [57]. This finding suggests that cytokine profiling could be a promising strategy for identifying at-risk patients.

Additionally, genomic and immunologic markers are being integrated into machine learning models to predict and mitigate the risk of irAEs (Table 3). For instance, a study developed a machine learning model using clinical data, genomics, and immune markers to predict the likelihood of irAEs in melanoma patients treated with nivolumab [58]. This approach has the potential to help clinicians tailor treatment plans, balancing the need for effective tumor control with the risk of harmful immune toxicity.

Efforts are also underway to develop pre-emptive strategies, such as prophylactic immunosuppressants and optimized dosing regimens, to manage irAEs while maintaining therapeutic efficacy. Understanding the mechanisms behind these toxicities could enable clinicians to mitigate adverse effects without compromising treatment outcomes.

### 5.3. Cost and Accessibility

Despite the promising potential of genomics-driven immunotherapy, the high costs associated with advanced molecular diagnostics and ICIs remain significant barriers to widespread access, especially in low- and middle-income countries. Next-generation sequencing (NGS), which is crucial for identifying TMB and other biomarkers, requires expensive equipment and expertise, limiting its availability [59]. In high-income countries, insurance coverage and cost-effectiveness are major concerns, as the costs of therapies like pembrolizumab can be prohibitive for many patients.

To address these challenges, biomarker-driven strategies have been proposed to ensure that only patients most likely to benefit from expensive therapies receive them. For instance, testing for PD-L1 expression and TMB can help stratify patients, ensuring that ICIs are used more efficiently. However, the lack of standardized testing methods and thresholds complicates their clinical application. A recent study demonstrated the variability in TMB testing across different laboratories, which impacts the reliability of these biomarkers as predictive tools [58]. Efforts to standardize these tests are underway, with the goal of making them more accessible and cost-effective.

In addition, the development of biosimilar drugs—chemically similar versions of expensive biologics—holds promise for reducing the financial burden of immunotherapy. Biosimilars like avelumab (an anti-PD-L1 antibody) are currently being evaluated for their effectiveness and affordability in comparison to branded therapies.

Moreover, the expansion of decentralized clinical trial models and digital health technologies offers new pathways to improve access to genomics-driven immunotherapy. AI-powered diagnostic tools, telemedicine platforms, and remote monitoring technologies could help bridge gaps in care by providing access to cutting-edge treatments for underserved populations. A recent pilot study explored the use of telemedicine to manage cancer patients in rural settings, demonstrating its potential to increase access to specialized care while reducing costs [60].

## 6. Future Directions

### 6.1. Integrating Multi-Omics for Personalized Immunotherapy

The future of cancer treatment lies in the integration of multi-omics data, including genomics, transcriptomics, proteomics, and metabolomics. Combining these layers of biological information offers the potential to better predict responses to immunotherapy and tailor treatments to the individual patient. A study demonstrated that integrating multi-omics approaches could uncover patient-specific biomarkers that predict immunotherapy outcomes in non-small-cell lung cancer (NSCLC) [61]. This suggests that the integration of multi-omics could similarly revolutionize the treatment landscape for GU cancers.

Spatial multi-omics, a cutting-edge technique that allows the simultaneous analysis of multiple molecular layers within tissue samples, holds particular promise in oncology. This method helps researchers understand how tumor and immune cells interact at the single-cell level within the TME. In GU cancers, where the TME plays a critical role in immune evasion, spatial multi-omics could reveal how heterogeneity within the tumor and immune cells affects immunotherapy response. A recent study utilized spatial transcriptomics to uncover immune escape mechanisms in bladder cancer, offering new insights into tumor–immune cell interactions and strategies for overcoming resistance to ICIs [62]. The incorporation of epigenomic data, which reveals the dynamic regulation of gene expression, and microbiome profiles, which influence immune responses, will enhance our understanding of the TME and facilitate the development of more personalized immunotherapies [63].

Additionally, CRISPR-based screening has become a powerful tool for functional genomics, enabling the identification of novel therapeutic targets. A landmark study applied CRISPR screening to identify genetic vulnerabilities, providing new avenues for combinatorial therapy [55]. As these technologies advance, they will become integral in optimizing immunotherapy strategies for GU cancer patients.

### 6.2. Exploring Novel Combination Therapies

Combination immunotherapy is one of the most promising strategies for overcoming resistance and enhancing the efficacy of treatment. While ICIs have proven effective in a variety of cancers, combining them with other therapeutic modalities may further boost their potential. For instance, the combination of ICIs with targeted therapies or epigenetic drugs has garnered interest for its ability to modify the tumor microenvironment and enhance immune cell infiltration. A study found that combining epigenetic modulators with ICIs in prostate cancer resulted in improved tumor regression and long-term survival [64]. This highlights the value of altering the epigenetic landscape to potentiate the effects of an immune checkpoint blockade.

The strategic sequencing of therapies is also critical. Researchers are investigating whether combination treatments work better when administered simultaneously or in a specific sequence. Studies such as the KEYNOTE-522 trial, which combined pembrolizumab with chemotherapy for triple-negative breast cancer, have provided important insights into optimal treatment sequencing.

**Figure 4:**
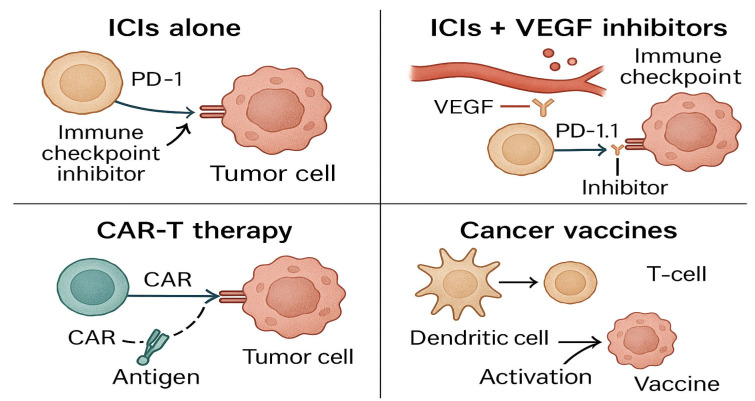
**Combination immunotherapy strategies compare** different combination approaches—such as ICIs (immune checkpoint inhibitors) with VEGF (vascular endothelial growth factor) inhibitors, CAR-T (chimeric antigen receptor T-cell), and cancer vaccines—used to enhance immune response in GU cancers. Created with Official Adobe Photoshop—Photo & Design Software.

The potential of oncolytic viruses and bispecific antibodies is also being explored to enhance immune activation. The combination of CAR-T-cell therapy with ICIs is another exciting approach, with early-phase trials indicating that this pairing could significantly improve anti-tumor responses while minimizing side effects. These combination therapies aim to address the evolving tumor landscape by tailoring treatments based on real-time tumor and immune responses, enhancing both efficacy and safety. Figure 4 provides a visual overview of current combination immunotherapy strategies.

### 6.3. Artificial Intelligence-Driven Precision Oncology

AI is rapidly becoming a key player in personalized cancer therapy, particularly in genomics-driven immunotherapy. The ability of AI to analyze vast datasets, such as multi-omics profiles and clinical records, offers the potential to revolutionize biomarker discovery and treatment decision-making. A groundbreaking study demonstrated how deep learning algorithms can predict cancer outcomes with a high degree of accuracy, using clinical data and digital pathology slides [65]. These AI-driven models could greatly enhance personalized treatment plans by offering more precise risk assessments and treatment recommendations.

AI is also being employed to predict drug resistance, a critical issue in immunotherapy. In a study, machine learning models were trained to anticipate which patients would develop resistance to ICIs, enabling clinicians to proactively adjust therapy regimens [66]. The integration of AI with radiomics, which involves extracting quantitative data from medical images, offers new avenues for monitoring disease progression and predicting treatment responses in real time.

**Figure 5:**
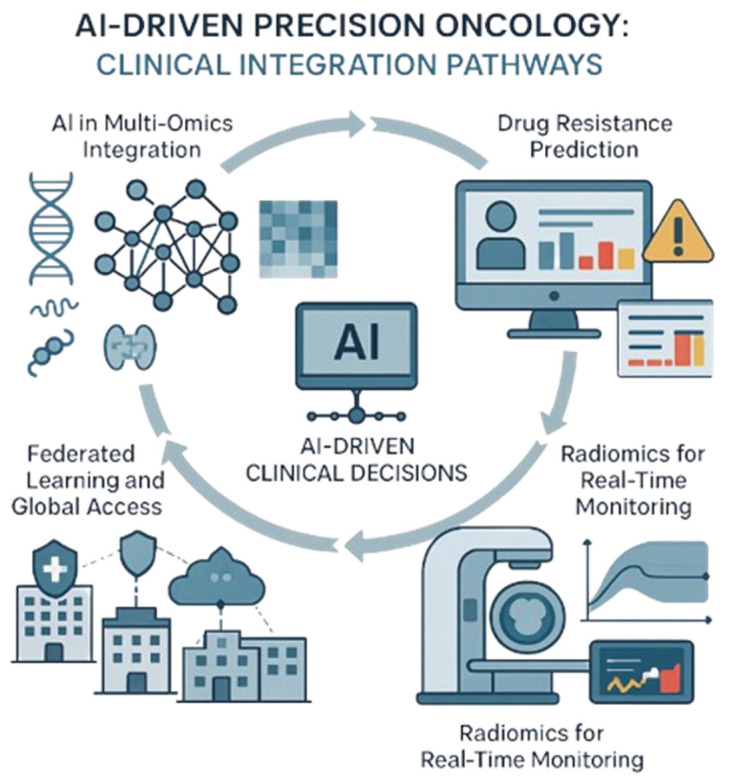
**AI-driven precision oncology: Clinical integration pathways.** This illustration shows how advanced tools help doctors personalize cancer treatment by combining different data types, tracking progress, predicting challenges, and securely sharing insights across hospitals. Created with Official Adobe Photoshop—Photo & Design Software.

To visually support these concepts, Figure 5 illustrates the diverse applications of AI in precision oncology. It highlights four interconnected clinical integration pathways: AI-powered multi-omics integration for therapy outcome prediction, machine learning-based drug resistance forecasting, radiomics for real-time monitoring, and federated learning frameworks that enable secure, collaborative AI model training across institutions.

Moreover, advancements in federated learning and synthetic data modeling are enhancing the security, transparency, and scalability of AI applications in oncology [67]. These innovations are critical for expanding access to precision oncology, particularly in resource-limited settings, where centralized data models might otherwise be difficult to implement.

### 6.4. Microbiome and Immune System Interactions

Recent studies have revealed the profound influence of the microbiome on cancer treatment outcomes. Specific bacterial species in the gut microbiota have been shown to enhance or hinder responses to immunotherapy, particularly ICIs. A study found that a diverse microbiome was associated with improved response rates to pembrolizumab in melanoma patients. These findings suggest that gut bacteria may influence systemic immunity and could be leveraged to optimize immunotherapy outcomes.

Emerging research is exploring microbiome-based therapies to modulate the gut environment and enhance anti-tumor immunity. Probiotics, dietary interventions, and fecal microbiota transplants are being investigated as potential strategies to create a gut environment that supports immune activation. A promising study showed that the oral administration of specific probiotics in mice enhanced the efficacy of PD-1 blockade in colon cancer, suggesting the potential of microbiome modulation as an adjunct to immunotherapy [68].

Furthermore, synthetic biology is enabling the engineering of bacteria to produce immune-activating molecules directly in the gut. This innovative approach could potentially reshape the microbiome to better support anti-tumor immunity, improving the efficacy of immunotherapies. As the field progresses, integrating microbiome analysis with other biological data, such as immune gene expression or cytokine levels, will allow for the development of personalized interventions aimed at optimizing immune responses [69].

### 6.5. Advancements in Non-Invasive Biomarkers

Non-invasive biomarkers are becoming essential tools for monitoring cancer progression and predicting treatment outcomes. Liquid biopsy technologies, which analyze circulating tumor DNA (ctDNA), exosomes, or tumor cells in the blood, offer a less invasive alternative to traditional tissue biopsies. A study demonstrated that ctDNA analysis could detect mutations and monitor treatment responses in real time, providing valuable insights into tumor dynamics [70]. Liquid biopsies hold great promise for the early detection of relapse and real-time monitoring of therapy efficacy, which is critical for adjusting treatment strategies and improving patient outcomes.

In addition, wearable biosensors are being developed to monitor physiological and molecular changes during treatment. These biosensors, often coupled with AI algorithms, could provide continuous, real-time data on patient health, enabling more personalized and adaptive treatment regimens. The integration of AI with liquid biopsy technologies could lead to high throughput, fully automated platforms capable of analyzing multiple biomarkers simultaneously. These platforms would enable clinicians to make quicker, data-driven decisions and improve patient outcomes by providing personalized treatment recommendations based on real-time data.

## 7. Conclusions

The integration of genomic research with immunotherapy has ushered in a transformative era for the treatment of GU cancers. The ability to tailor treatments based on individual tumor profiles has significantly advanced the field of precision oncology, providing new hope for more effective and personalized therapies. Through genomic profiling, key biomarkers have been identified that enable clinicians to predict patient responses to immunotherapy, leading to improved outcomes and reduced toxicity. However, challenges remain in fully utilizing the potential of these innovations. The ongoing refinement of predictive biomarkers and the development of more accurate patient-specific treatment plans will be essential to maximizing the benefits of genomic-driven immunotherapy.

At the same time, broadening access to these cutting-edge treatments remains a critical challenge. While the promise of precision medicine is immense, the high costs and complexities associated with genomic testing and immunotherapy limit its availability to many patients, especially in low-resource settings. To overcome these barriers, there is a pressing need for more affordable and scalable approaches. The convergence of multi-omics technologies, artificial intelligence, and microbiome profiling has the potential not only to enhance the efficacy of immunotherapies but also to make these innovations more accessible and applicable across diverse patient populations.

As we look toward the future, it is crucial to focus on developing cost-effective solutions for advanced immunotherapy treatments, particularly for underserved populations. Advances in biomanufacturing, the automation of cell therapy production, and the optimization of gene-editing technologies may pave the way for making these therapies more widely available in clinical settings. Furthermore, leveraging real-world data and adopting more flexible, inclusive clinical trial designs will be key to translating research breakthroughs into therapies that benefit a broader range of patients.

Collaboration across disciplines—spanning scientific research, clinical practice, regulatory frameworks, and industry—will be paramount to driving progress. By embracing systems biology, computational modeling, and patient-centered research, the field of GU cancer care can continue to evolve. Only through these integrated efforts can we ensure that the future of cancer treatment is not just innovative but also equitable and impactful, delivering meaningful change to global healthcare systems and improving the lives of patients worldwide.

The promise of genomics-driven immunotherapy lies not only in its technological potential but also in its ability to make personalized, life-saving treatments accessible to all, ushering in a new era of cancer care that is as inclusive as it is transformative.

## Figures and Tables

**Table 1:** Genomic alterations in genitourinary cancers.

Cancer Type	Key Genetic Alterations	Molecular Subtypes	Clinical Implications
Prostate cancer	ETS fusion, SPOP mutation, TP53 loss, BRCA1/2 mutations	Luminal A, luminal B, basal	AR-targeted therapy, PARP inhibitors
Renal cell carcinoma	VHL loss, PBRM1 mutation, SETD2 loss	ccRCC, pRCC, chRCC	VEGF inhibitors, ICIs
Bladder cancer	FGFR3 mutation, TP53 mutation, ERBB2 amplification	Luminal, basal	FGFR inhibitors, ICIs
c	KIT mutation, KRAS mutation (more frequent in seminoma), Chromosomal gains/losses	Seminoma, non-seminoma	Chemotherapy, emerging immunotherapy

ETS (E26 transformation-specific), SPOP (speckle-type POZ protein), VHL (Von Hippel–Lindau), FGFR3 (Fibroblast Growth Factor Receptor 3), ERBB2 (Erythroblastic Oncogene B2, also known as HER2), ccRCC (clear cell renal cell carcinoma), pRCC (papillary renal cell carcinoma), chRCC (chromophobe renal cell carcinoma), AR (androgen receptor), PARP (poly ADP-ribose polymerase), and VEGF (vascular endothelial growth factor). This table outlines major genetic changes, molecular subtypes, and related treatment strategies in prostate, kidney, bladder, and testicular cancers. Understanding these alterations helps classify tumors more precisely and tailor therapies to individual patients. These insights also pave the way for targeted and precision oncology in GU malignancies.

**Table 2:** FDA-approved and investigational immunotherapies in genitourinary cancers.

Drug Name	Target	Cancer Type	Clinical Trial/Approval Status
Pembrolizumab	PD-1	Bladder, kidney	FDA-Approved (KEYNOTE-045)
Nivolumab	PD-1	RCC, bladder	FDA-Approved (CheckMate 214)
Atezolizumab	PD-L1	Bladder	FDA-Approved
Ipilimumab + Nivolumab	CTLA-4 + PD-1	RCC	FDA-Approved
CAR-T therapy (Experimental)	TCR-based	Prostate	Ongoing Trials

FDA (Food and Drug Administration), TCR (T-cell receptor), and RCC (renal cell carcinoma).

**Table 3:** Biomarkers for immunotherapy response in genitourinary cancers.

Biomarker	Significance	Detection Method	Clinical Relevance
PD-L1	Predictive response to ICIs	Immunohistochemistry (IHC)	Commonly used for patient stratification
Tumor Mutational Burden (TMB)	Higher values linked to better ICI outcomes	NGS-based sequencing	Promising but lacks standardized thresholds
MSI/dMMR	High MSI tumors respond well to immunotherapy	PCR, IHC	FDA-approved biomarker in multiple cancer types
ctDNA (Liquid Biopsy)	Enables non-invasive monitoring of tumor dynamics	NGS-based liquid biopsy	Under active investigation for clinical use

MSI (microsatellite instability), dMMR (deficient mismatch repair), ctDNA (circulating tumor DNA), ICIs (immune checkpoint inhibitors), NGS (next-generation sequencing), and FDA (Food and Drug Administration). This provides an overview of important biomarkers used to guide immunotherapy, including detection methods and their clinical relevance in GU cancers. Reliable biomarkers are critical for identifying which patients are most likely to benefit from immunotherapy. Ongoing research continues to refine these markers to improve outcomes and avoid unnecessary treatment.

## Data Availability

Not applicable.

## Abbreviations

This review includes several key abbreviations: **GU** refers to genitourinary, a term encompassing cancers of the prostate, kidney, bladder, and testes. **ICIs** stand for immune checkpoint inhibitors, which target proteins like **PD-1** (programmed death-1), **PD-L1** (programmed death ligand-1), and **CTLA-4** (cytotoxic T-lymphocyte-associated protein 4) to reactivate immune responses. **CAR-T** (chimeric antigen receptor T-cell) and **TIL** (tumor-infiltrating lymphocyte) therapies represent forms of adoptive cell transfer. **TCR** refers to T-cell receptor-engineered cells. The **AR** (androgen receptor) pathway and **ADT** (androgen deprivation therapy) are pivotal in prostate cancer. Genetic profiling often utilizes **NGS** (next-generation sequencing), and **ctDNA** (circulating tumor DNA) analysis is increasingly used for non-invasive diagnostics. **RCC** (renal cell carcinoma) and its subtype **ccRCC** (clear cell renal cell carcinoma) are commonly treated with **VEGF** (vascular endothelial growth factor) inhibitors. Biomarkers such as **TMB** (tumor mutational burden), **MSI** (microsatellite instability), and **dMMR** (deficient mismatch repair) help predict ICI response. The **TME** (tumor microenvironment) includes immunosuppressive elements like **MDSCs** (myeloid-derived suppressor cells), **Tregs** (regulatory T-cells), and **TAMs** (tumor-associated macrophages). Large-scale projects like **TCGA** (The Cancer Genome Atlas) and **GWAS** (genome-wide association studies) have deepened genomic understanding. **AI** (artificial intelligence) supports multi-omics data integration, aiding in **precision oncology**. **PARP** (poly ADP-ribose polymerase) inhibitors target DNA repair deficiencies, while metabolic mutations involve enzymes such as **FH** (fumarate hydratase) and **SDH** (succinate dehydrogenase). Also discussed are **mRNA** (messenger RNA) vaccines and **HIF** (hypoxia-inducible factor) pathways in RCC.
